# Synaptosomes: new vesicles for neuronal mitochondrial transplantation

**DOI:** 10.1186/s12951-020-00748-6

**Published:** 2021-01-06

**Authors:** Pasquale Picone, Gaetana Porcelli, Celeste Caruso Bavisotto, Domenico Nuzzo, Giacoma Galizzi, Pier Luigi San Biagio, Donatella Bulone, Marta Di Carlo

**Affiliations:** 1 Istituto per la Ricerca e l’ Innovazione Biomedica (IRIB) CNR, via U. La Malfa 153, 90146 Palermo, Italy; 2 Istituto di Biofisica (IBF) (sez. Palermo) CNR, via U. La Malfa, 153, 90146 Palermo, Italy; 3 Dipartimento di Biomedicina, Neuroscienze, e Diagnostica Avanzata (BIND) (Sez. Anatomia Umana), Università di Palermo, via del Vespro 129, 90127 Palermo, Italy; 4 Istituto Euro-Mediterraneo di Scienze e Tecnologie (IEMEST), via M. Miraglia, 20, 90139 Palermo, Italy

**Keywords:** Synaptosomes, Mitochondria, Neurodegeneration, Delivery system, Mitochondrial transplantation

## Abstract

**Background:**

Mitochondrial dysfunction is a critical factor in the onset and progression of neurodegenerative diseases. Recently, mitochondrial transplantation has been advised as an innovative and attractive strategy to transfer and replace damaged mitochondria. Here we propose, for the first time, to use rat brain extracted synaptosomes, a subcellular fraction of isolated synaptic terminal that contains mitochondria, as mitochondrial delivery systems.

**Results:**

Synaptosome preparation was validated by the presence of Synaptophysin and PSD95. Synaptosomes were characterized in terms of dimension, zeta potential, polydispersity index and number of particles/ml. Nile Red or CTX-FITCH labeled synaptosomes were internalized in LAN5 recipient cells by a mechanism involving specific protein–protein interaction, as demonstrated by loss of fusion ability after trypsin treatment and using different cell lines. The loading and release ability of the synaptosomes was proved by the presence of curcumin both into synaptosomes and LAN5 cells. The vitality of mitochondria transferred by Synaptosomes was demonstrated by the presence of Opa1, Fis1 and TOM40 mitochondrial proteins and JC-1 measurements. Further, synaptosomes deliver vital mitochondria into the cytoplasm of neuronal cells as demonstrated by microscopic images, increase of TOM 40, cytochrome c, Hexokinase II mitochondrial proteins, and presence of rat mitochondrial DNA. Finally, by using synaptosomes as a vehicle, healthy mitochondria restored mitochondrial function in cells containing rotenone or CCCp damaged mitochondria.

**Conclusions:**

Taken together these results suggest that synaptosomes can be a natural vehicle for the delivery of molecules and organelles to neuronal cells. Further, the replacement of affected mitochondria with healthy ones could be a potential therapy for treating neuronal mitochondrial dysfunction-related diseases.
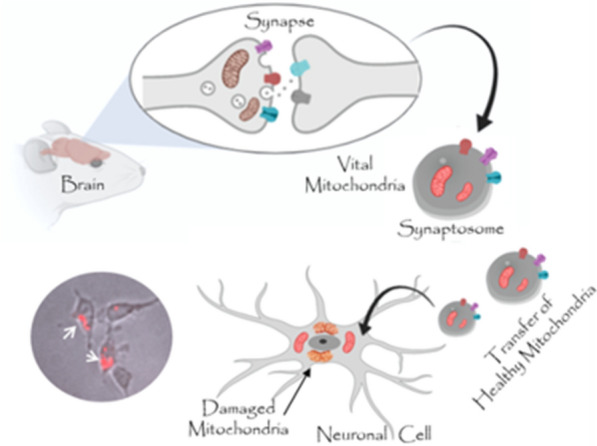

## Background

Neurodegenerative diseases (NDs) are debilitating age-related disorders characterized by progressive loss of structural and functional neurons. It has been estimated that approximately 30 million people worldwide are affected by NDs and the number is expected to exceed 150 million in 2050 [1]. The lack of any effective treatment makes these pathologies a significant public health issue. NDs are characterized by the accumulation of aggregates of misfolded proteins in specific areas of the brain and they include Alzheimer's disease (AD), Parkinson's disease (PD), and Huntington's disease (HD) [1]. These pathologies are known to be multifactorial disorders, but one of the most accepted assumptions is that mitochondrial dysfunction is a critical factor in their onset and progression [1–4]. Mitochondria are the primary source of energy metabolism and play a relevant role in several cell functions such as calcium homeostasis, reactive oxygen species (ROS) production, cell survival and proliferation, control of apoptosis and autophagy [3]. Mitochondria are dynamic organelles capable of changing size, shape, and position according to the cells' physiological needs. They move along microtubules within the cell, providing the energy for the different cells' activities, including the synaptic ones. Mitochondrion wellness is crucial for cellular homeostasis and its impairment is linked to several neurodegenerative diseases. Mitochondrial dysfunction is associated with altered antioxidant defence or excessive ROS generation. The use of natural antioxidants as potential therapeutic molecules for the prevention and treatment of NDs has been largely explored [4, 5] Antioxidants such as resveratrol, green tea polyphenol, epigallocatechin gallate (EGCC) or ferulic acid were found to exert beneficial effects both on in vitro and in vivo models of NDs [6–9]. Although quite controversial, some studies in humans report that the administration of high-dose combined vitamin E and vitamin C is associated with a slowed progression of PD. Further, the beneficial effect can be improved by encapsulating the antioxidant molecules into nanoparticles capable of enhancing drug transport through the Blood Brain Barrier (BBB) [10, 11]. Studies in vitro reported that insulin could prevent mitochondrial oxidative stress and apoptosis by inhibiting the PI3K/AKT cell survival signaling pathway, similarly to what was also observed for antioxidant molecules [12, 13]. Recent evidence has demonstrated that insulin delivery to the brain can be an effective pharmacological therapy for some neurodegenerative pathologies, and the intranasal route can further increase the efficacy and safety of the treatment [14].

Unfortunately, all these strategies to contrast mitochondrial stress are not considered yet effective prevention or therapy for NDs, and the possibility to replace damaged mitochondria results in being extremely attractive.

Mitochondria have a life cycle in which mitochondrial dynamics and mitophagy contribute to quality control. The dynamics of mitochondria is regulated by fusion and fission events. These mechanisms are controlled by specific proteins such as mitofusin 1 (MNF1) and optic atrophy protein1 (OPA1) for fusion, and dynamin-related protein-1 (Drp1) and fission1 (FIS1) for fission [15]. When mitochondria are damaged, the organelles can be recovered by fusing with healthy mitochondria or eliminated by mitophagy. However, when damaged mitochondria cannot be replaced or restored, the possibility to transfer healthy mitochondria from one cell to another represents an attractive therapeutic strategy. Currently, the transfer of "alive" mitochondria into injured cells takes the name of mitochondrial transplantation and it is becoming a popular approach for the treatment of several diseases, including NDs [16–19].

Some strategies such as direct microinjection of isolated mitochondria, cell-mediated transfer utilizing tunneling nanotubes, vesicle- or liposome-mediated delivery, and systemic delivery have been evaluated to ameliorate mitochondria uptake and transplantation efficiency [19]. Nevertheless, adverse effects such as inflammatory or immune response due to the introduction of cellular elements with mitochondria can interfere with the transplantation efficacy [19]. Instead, an increased incorporation efficiency was observed when isolated mitochondria from a healthy individual were conjugated with the carrier peptide Pep-1 [20]. However, delivery approaches have to be optimized based on the tissue or cell type.

Synaptosomes are a subcellular fraction isolated from synaptic terminals that can be prepared by homogenization and gradient centrifugation of brain tissue. The term "synaptosome" was coined by Whittaker (1964) [21], who studied the localization of neurotransmitters and the functional components of the synapses. Synaptosomes contain numerous synaptic vesicles and mitochondria and are considered a relevant model system for studying human synaptic dysfunction in NDs [22]. Although the use of cells or cell membrane-based drug delivery systems has been investigated [23, 24], the possibility of using a subcellular structure for organelle delivery has not been studied yet.

For the first time, we propose using a synaptosome-based mitochondria delivery system to transfer healthy mitochondria to neuronal target cells. Furthermore, we evaluated the possibility of replacing CCCp- or rotenone-damaged mitochondria in neuronal cells by synaptosomes transplantation.

## Results

### Isolation and characterization of rat synaptosomes

**Fig. 1:**
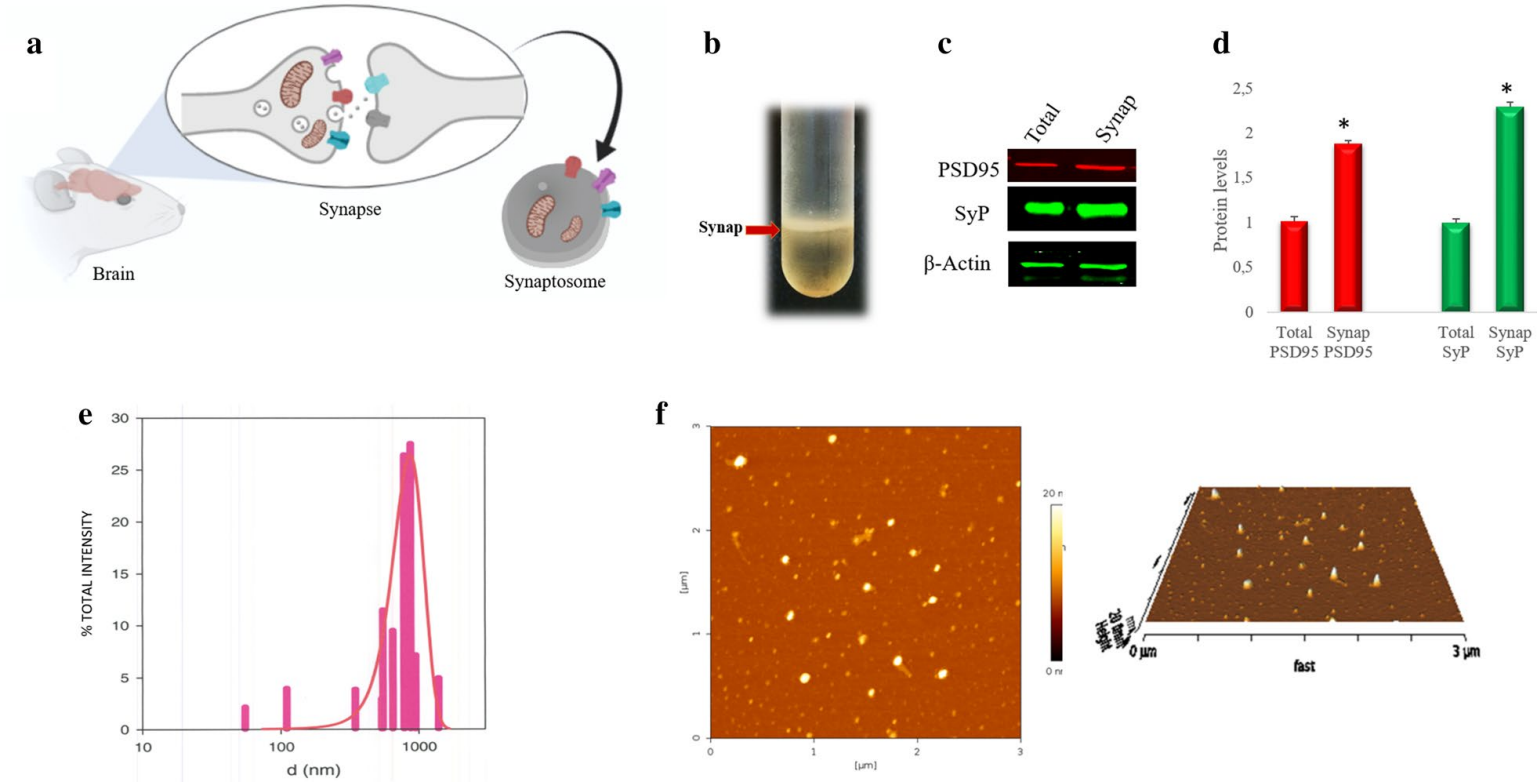
Synaptosomes isolation and characterization. **a** Representation of synaptosomal particles with pre- and post-synaptic areas evidenced. **b** Synaptosomal band (red arrow) collected at sucrose interface after ultracentrifugation in sucrose fractionation. **c** Western blot of proteins extracted from total homogenate (Total) and synaptosomal fraction (Synapt) incubated with anti-PSD95 (PSD95) and anti- Synaptophysin (SyP). **d** Relative quantification of the immunoreactive bands. The uniformity of gel loading was confirmed by using β-actin as a standard. *P < 0.05, vs total homogenate (Total). **e** Intensity weighted size distribution functions of the synaptosomes extracted. **f** AFM images of the synaptosomes extracted

Synaptosomes, extracted from the pre- and post-synaptic components (Fig. 1a), were isolated by sucrose density gradient ultracentrifugation of the fresh rat brain homogenate. The presence of synaptosomes in the collected band (Fig. 1b) was ascertained by detecting with Western blot experiments the presence of two specific pre-synaptic and post-synaptic markers, Synaptophysin and PSD95, respectively (Fig. 1c). A significant content of these two proteins was found in the collected band with respect to the total homogenate (Fig. 1c, d), confirming the fractionation procedure's success. By Dynamic Light Scattering (DLS) measurements, the synaptosomes' size was determined to range between 0.5 and 1.5 µm with a mean diameter of about 800 nm (Fig. 1e). AFM images (Fig. 1f) demonstrated the typical synaptosomes features, confirming the heterogeneity of the preparation. The synaptosomes fraction was also characterized in terms of zeta potential, polydispersity index (PDI), concentration. The values are reported in Table 1.

**Table 1:** Concentration (particles/ml), zeta potential and polydispersity index of extracted synaptosomes

Synaptosome parameters	
[Particles/m1]	5.1 × 10^9^ ± 0.7 × 10^9^
Zeta Potential [mV]	− 43.5 + 5
polydispersity	0.307

### Synaptosomes cell interaction and uptake

**Fig. 2:**
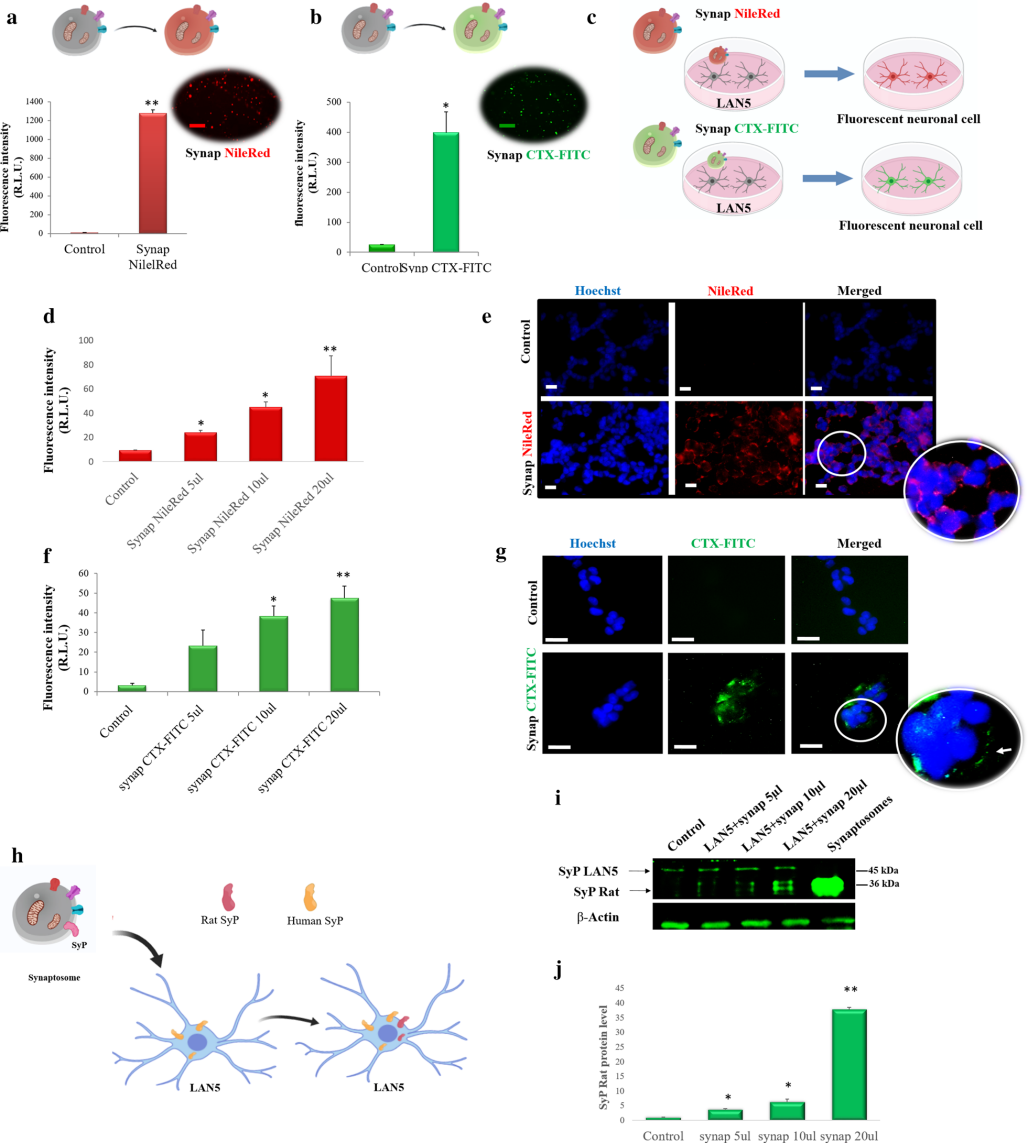
Synaptosomes can interact with cells. **a** Histogram of fluorescence intensity and fluorescence image of synaptosomes stained with Nile Red (Synap Nile Red) and related cartoon. **b** Histogram of fluorescence intensity and fluorescence image of synaptosomes stained with CTX-FITC (Synap CTX-FITC) and related cartoon. **c** Cartoon of neuronal cells incubated with synaptosomes stained with Nile Red or CTX-FITC. **d** Histogram of fluorescence intensity of LAN5 cells incubated with different doses of synaptosomes labeled with Nile Red (Synap Nile Red 5–10 and 20 µl). **e** Representative fluorescence images of Synap NileRed and LAN5 cells interaction, nuclei stained with Hoechst 33342. **f** Fluorescent intensity histogram of LAN5 cells incubated with different doses of synaptosomes labeled with CTX-FITC (Synap CTX-FITC 5–10 and 20 µl). **g** Representative fluorescence images of Synap CTX-FITC and LAN5 cells interaction. Nuclei were stained with Hoechst 33342. **h** Schematic representation of the localization of Rat synaptophysin (Rat SyP) (red) and Human synaptophysin (Human SyP) (yellow) before and after administration of synaptosomes in LAN5 cell. **i** Western blot analysis of proteins extracted from LAN5 cells untreated (Control) or treated with different doses of synaptosomes (5–10–20 µl) (Lan5 + synap), and from synaptosomes incubated with anti-Synaptophysin (SyP). **j** Relative quantification of Rat synaptophysin (Rat SyP) immunoreactive band (36 kDa). The uniformity of gel loading was confirmed by using β-actin as a standard. *P < 0.05, **P < 0.02 vs Control. Scale bar 20 µm. Synap 5–10 and 20 µl; correspond to concentration of 2.5 × 10^7^; 5.1 × 10^7^; 10.2 × 10^7^ particles/100 µl respectively)

Synaptosomes are membranous sacs composed of a lipid bilayer, including lipid rafts [25] and synaptic components. Intending to use them as delivery systems, firstly, we investigated if synaptosomes were toxic to LAN5 neuronal cells (Additional file 1: Fig. S1). Synaptosomes were incubated with the membrane staining Nile Red (red), or with the lipid raft staining CTX (green). Results from fluorescence measurements (Fig. 2a, b) showed that both the dyes were suitable for staining the synaptosomes and, therefore, as vesicle tracing. LAN5 cells were incubated with different amounts of synaptosomes stained with Nile Red (red) or CTX-FITC (green), and the fluorescence signal was measured to evaluate cellular uptake of synaptosomes (Fig. 2c). The fluorescence intensity was observed to increase in a dose-dependent manner for both the tracker dyes (Fig. 2d, f). The interaction between synaptosomes and LAN5 was also visualized by fluorescence microscopy inspection (Fig. 2e, g). LAN5 uptake of synaptosomes was furtherly evidenced by detecting the presence of rat synaptophysin, a presynaptic protein associated with synaptic vesicles and presynaptic membrane [26]. After administering different amounts of synaptosomes to LAN5 cells, the presence of the rat synaptophysin was investigated (Fig. 2h). Western blot experiments on LAN5 extracted confirmed the presence of a band of about 36 kDa corresponding to rat synaptophysin (Fig. 2i). This band intensity was higher at larger administered doses of synaptosomes (Fig. 2i, l). A higher band of 45 kDa with the same intensity, representing the human endogenous protein, was also observed (Fig. 2i). As the trypsin treatment used in protein extraction removes synaptosomes interacting with the cellular surface, it is reasonable that the detected rat synaptophysin was due to internalization of synaptosomes or fusion of membranes.

### Synaptosomes as a potential delivery system

**Fig. 3:**
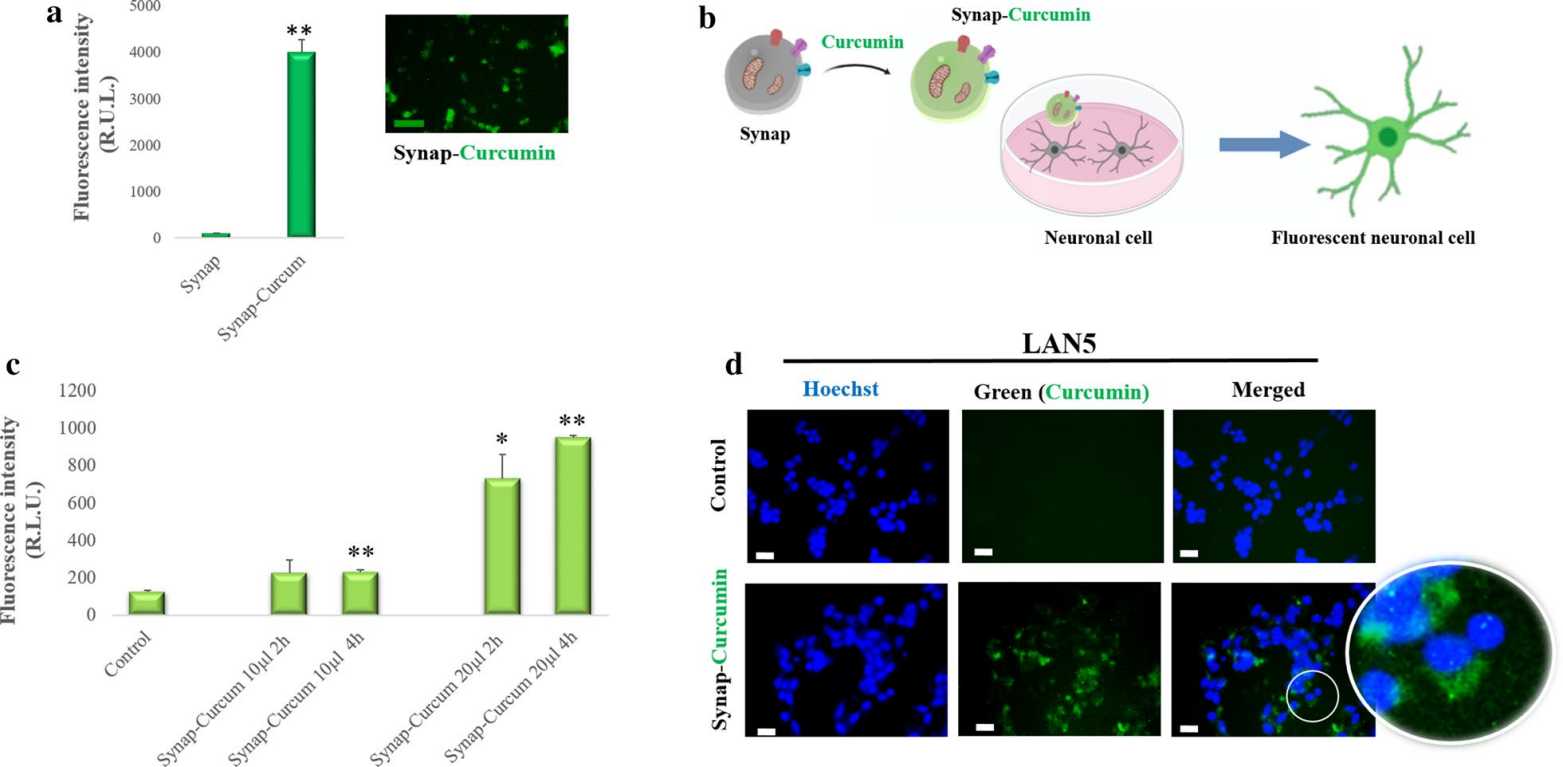
Synaptosomes are able to load and release curcumin. **a** Histogram of the fluorescence intensity and fluorescence image of synaptosomes loaded with Curcumin (Synap-Curcumin). **b** Schematic representation of LAN5 neuronal cell incubated with Synap-Curcumin. **c** Histogram of the fluorescence intensity of LAN5 cells incubated with different doses (10–20 µl, which correspond to concentration of 5.1 × 10^7^; 10.2 × 10^7^ particles/100 µl respectively) of Synap-Curcumin for 2 and 4 h. **d** Representative fluorescence images showing the interaction of Synap-Curcumin with LAN5 cells and control (not treated). Nuclei were stained with Hoechst 33342. *P < 0.05, **P < 0.02 vs Control. Scale bar 20 µm

The ability of synaptosomes to release the cargo was tested by using curcumin, a fluorescent compound derived from the rhizome of Curcuma longa. It had been shown that curcumin could be loaded into lipid nanosystems and internalized in neuronal cells [27]. After curcumin addition to synaptosomes (Fig. 3a) an increase of the fluorescence intensity was observed, indicating that synaptosomes were able to incorporate curcumin. Different amounts of curcumin-loaded synaptosomes (Synap-Curcumin) were then incubated with LAN5 cells for 2 and 4 h (Fig. 3b). An enhancement of fluorescence intensity was observed in the sample treated with 20 µl of curcumin-loaded synaptosomes, especially after 4 h of incubation (Fig. 3c). The results were confirmed by microscopy images (Fig. 3d). Green fluorescence was observed in the cellular body, confirming that the compound was released by the synaptosomes inside the cells (Fig. 3d).

### Synaptosomes-specific cell interaction

**Fig. 4:**
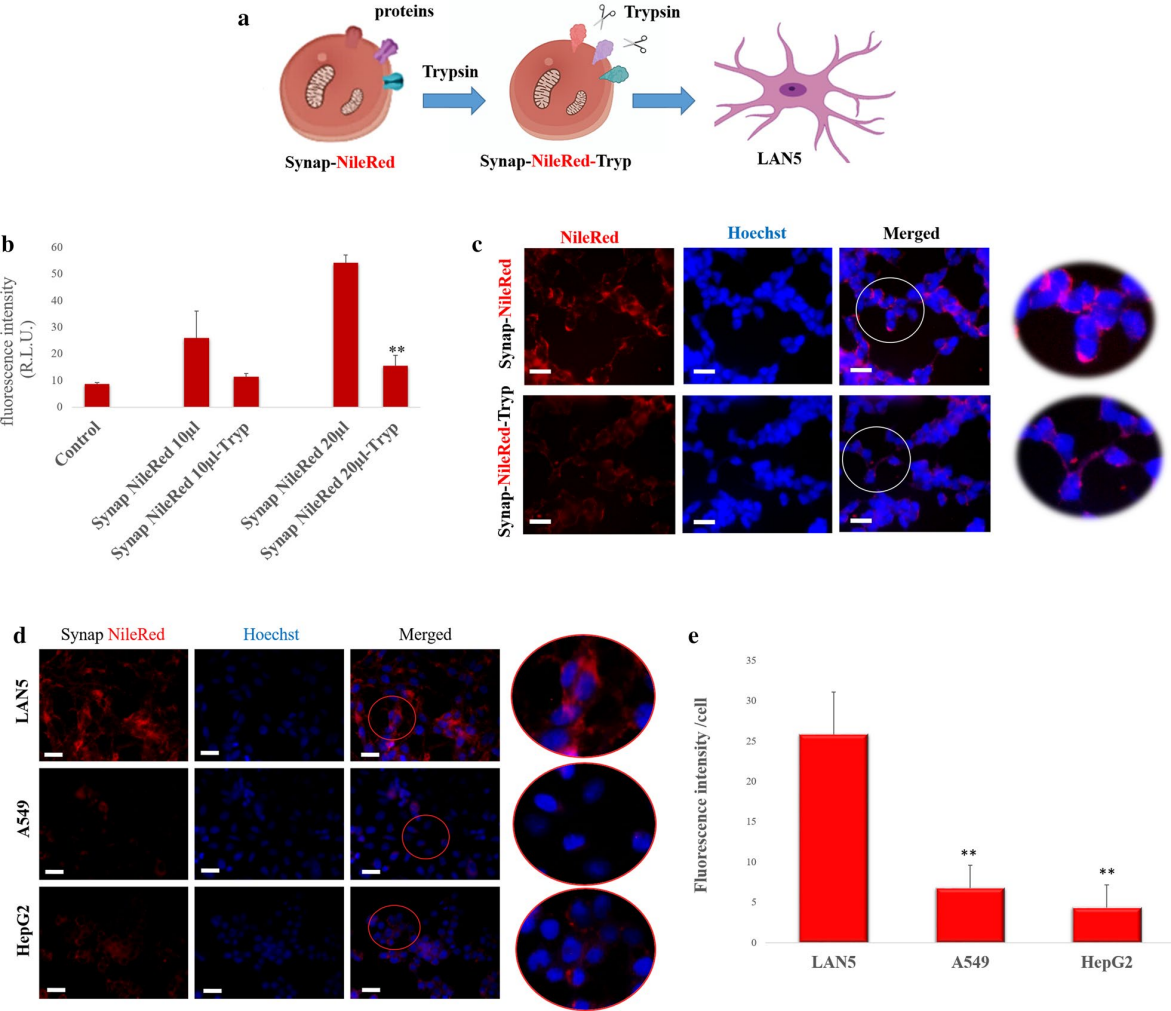
Synaptosomes–cell interaction depends on specific proteins. **a** Schematic representation of synaptosomes labeled with Nile Red and incubated with trypsin (Synap-NileRed-Tryp) before incubation with LAN5 cells. **b** Histogram of fluorescence intensity for LAN5 cells incubated with Synap-NileRed-Tryp and Synap-NileRed at different doses (10–20 µl, that correspond to concentration of 5.1 × 10^7^; 10.2 × 10^7^ particles/100 µl respectively) and for the relative control (untreated cells). **c** Representative fluorescence images for LAN5 cells incubated with Synap-NileRed and Synap-NileRed-Tryp. Nuclei were stained with Hoechst 33342. **P < 0.02. **d** Representative fluorescence images of LAN5, A549 and HepG2 cells after incubation with Nile Red labeled Synaptosomes (concentration of 10.2 × 10^7^ particles/100 µl) Nuclei were stained with Hoechst 33,342. **e **Histogram of the relative fluorescence intensity for the three types of cells. **P < 0.02 vs LAN5. Scale bar 20 µm

Nile red-stained synaptosomes were treated with two different amounts of trypsin (Fig. 4a) and incubated with LAN5 cells in order to inspect whether the contact between synaptosomes and the cell membrane is due to protein–protein and/or lipid-protein interaction. The fluorescence intensity for cells incubated with synaptosomes treated with trypsin was found lower than that for cells incubated with untreated synaptosomes (Fig. 4b). Microscopic images of Fig. 4c confirmed this result. Here, the red signal that indicates interaction between cells and synaptosomes is significantly lower in the case of trypsin treated synaptosomes. This outcome suggests that some proteins disrupted by the trypsin treatment could play a relevant role in synaptosomes cellular uptake. Furthermore, two amounts of Nile red-stained synaptosomes were administered to different cell lines, including neuronal (LAN5), epithelial (A549) and hepatic (HepG2) cells, to assess if cellular interaction and uptake of synaptosomes are neuron-specific. By microscopy imaging (Fig. 4d) and fluorescence measurements (Fig. 4e) we observed no significant fluorescence signal for both A549 and HepG2 cells, while fluorescence signals were detected in LAN5 cells. This evidence can suggest that specific neuronal proteins are necessary for synaptosomes-cell interaction and uptake.

### Synaptosomes transport vital mitochondria that can be cryopreserved

**Fig. 5:**
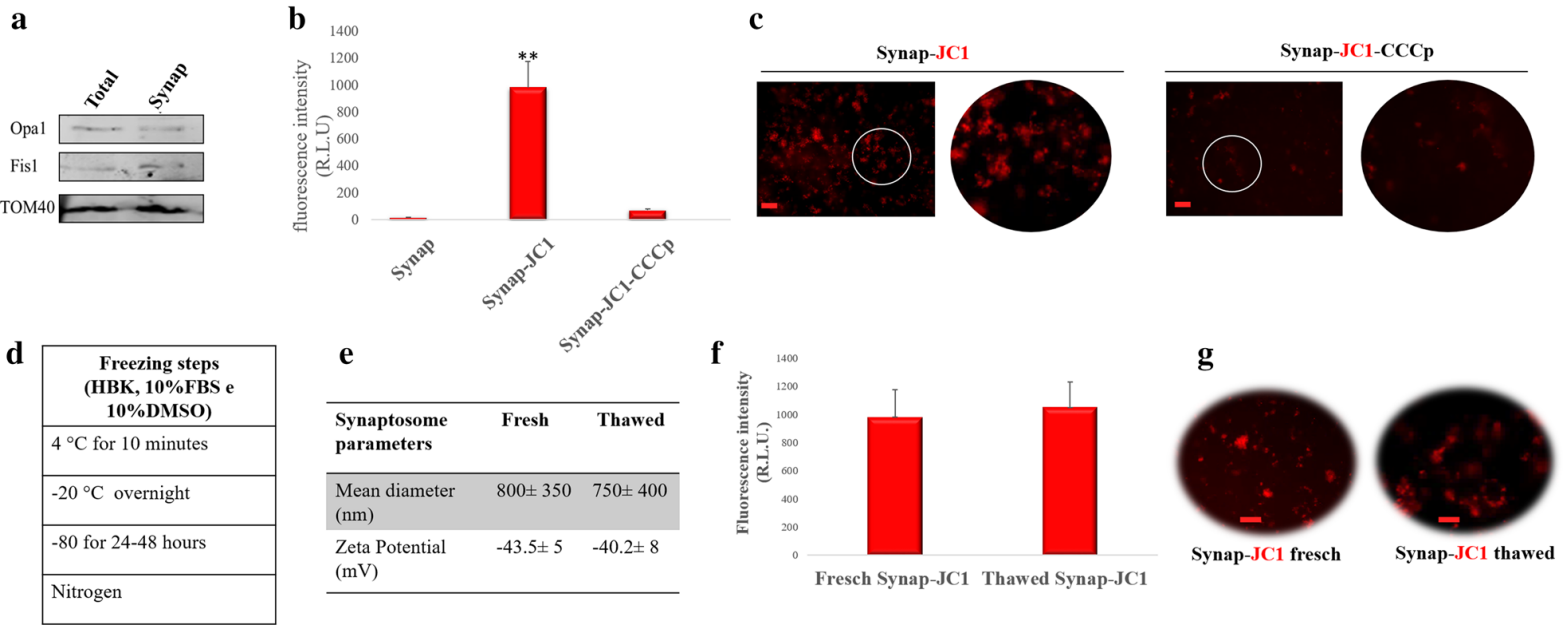
Synaptosomes contain vital mitochondria. **a** Western blotting analysis of proteins extracted from total homogenate (Total) and synaptosomal fraction (Synapt) incubated with antibodies against the mitochondrial proteins Opa1, Fis1 and TOM40. **b** Red fluorescence intensity for synaptosomes incubated with or without (Synap) JC1 and for synaptosomes treated with CCCp and incubated with JC-1 (Synap-JC1-CCCp) (positive control). **c** Representative JC1 fluorescence images of synaptosomes incubated with or without (Synap-JC1) CCCp. Cryopreservation of Synaptosomes **d** Schematic representation of the synaptosomes freezing procedure. **e** Table of the values of mean diameter and zeta potential of fresh and thawed synaptosomes. **f** Red fluorescence intensity of fresh (fresh Synap-JC1) and thawed (thawed Synap-JC1) synaptosomes incubated with JC-1. **g** Representative JC1 red fluorescence images of fresh and thawed synaptosomes incubated with JC-1. **P < 0.02 vs Control. Scale bar 20 µm

Synaptosomes contain synaptic vesicles and often one or more mitochondria. Mitochondria presence and viability in isolated synaptosomes were analyzed. The presence of mitochondrial specific proteins including the protein optic atrophy 1 (Opa1) (inner mitochondrial membrane protein), Fission 1 protein (Fis1) (outer mitochondrial membrane proteins) and Translocase of Outer Mitochondrial Membrane 40 (TOM40) in synaptosomal preparation was investigated by Western blotting analysis. The three proteins were detected in synaptosomal fractions, meaning that synaptosomes contain mitochondria (Fig. 5a). This result was also confirmed by staining synaptosomes with JC1, a fluorescent dye that emits red fluorescence when the mitochondrial membrane potential is high and mitochondria are healthy (Fig. 5b). To further validate the presence of mitochondria, synaptosomes were treated with CCCp, an inhibitor of oxidative phosphorylation. A drastic reduction of red fluorescence was observed in this case (Fig. 5b). Representative fluorescence images for all samples are shown in Fig. 5c. Thus, synaptosome preparation contains functional mitochondria.

Finally, we evaluated their physical–chemical properties and biological activity to test whether cryopreserved synaptosomes can be used for mitochondrial transplantation. Rat cerebral cortex synaptosomes preparation was frozen under controlled temperature steps and times (Fig. 5d). After thawing, physical parameters such as dimensions and zeta potential were measured and compared with those of fresh synaptosomes. No significant difference was detected (Fig. 5e). The viability of mitochondria, after freeze/thawing of synaptosomes, was assessed by JC-1 fluorescence assay. The results showed that the red and green fluorescence of the thawed synaptosomes was comparable to that of fresh synaptosomes (Fig. 5f, g), showing that despite the temperature variations, their functionality was preserved. Moreover, the synaptosomes' long-term storage up to 12 months did not influence the valuated synaptosomal parameters (data not shown). Thus, from the next experiments, we used freeze/thawed synaptosomes.

### Synaptosomes as mitochondrial delivery systems

We explored the possibility of using synaptosomes to transfer functional mitochondria in cells. There are various techniques for mitochondrial transplantation, but the opportunity to use synaptosomes as a delivery system has not been explored yet.

**Fig. 6:**
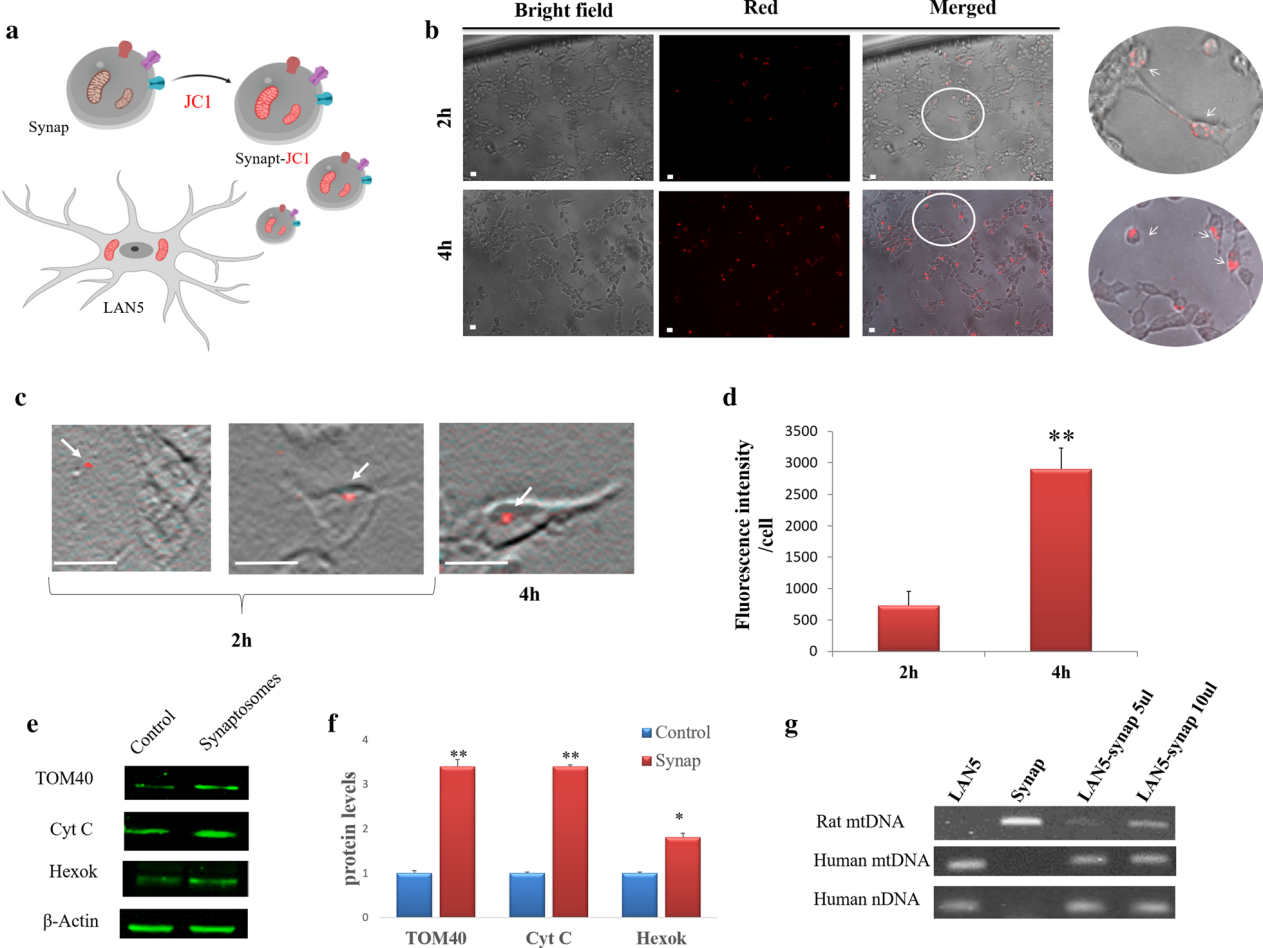
Synaptosomes can vehicle mitochondria **a** Cartoon of synaptosomes containing JC1-stained mitochondria (Synap-JC1) administered to LAN5 neuronal cells. **b** Representative bright field and red fluorescence images showing the interaction between Synap-JC1 and LAN5 cells after 2 or 4 h of incubation. **c** Representative dynamic imaging of Synap-C-1 at different times. **d** Histogram of the relative JC1 red fluorescence intensity for cell incubated with Synap-JC1 for 2 and 4 h (concentration of 10.2 × 10^7^ particles/100 µl). **e** Western blot of proteins extracted from LAN5 untreated (Control) or synaptosomes treated (Synap) and incubated with antibodies against mitochondrial proteins TOM40, Cytochrome C (Cyt C) and Hexokinase II (Hexok). **f** Relative quantification of the immunoreactive bands. The uniformity of gel loading was confirmed by using β-actin as standard. **g** PCR analysis of the mitochondrial DNA (mtDNA) specific for Human (Human mtDNA) and Rat (Rat mtDNA) in human LAN5 cells incubated with rat synaptosomes at different doses (5 and 10 µl, which correspond to concentration 2.5 × 10^7^; 5.1 × 10^7^ particles/100 µl respectively). Human nuclear DNA (Human nDNA) was used as reference. *P < 0.05, **P < 0.02 vs Control. Scale bar 20 µm

Synaptosomes containing JC1 stained mitochondria (Synap-JC1) were administrated to LAN5 cells and incubated for two different time intervals (Fig. 6a). After two hours of incubation, a light punctate red fluorescence was detected by fluorescence microscopy on LAN5 membranes, whereas after 4 h, an intense red fluorescence was observed in LAN5 cytoplasm, suggesting that cellular uptake of the synaptosomal-mitochondria occurred (Fig. 6b). Dynamic imaging simulation was performed to detect uptake and trafficking of Synap-JC-1 fully. As shown in Fig. 6c, after 2 h, some synaptosomes were approaching the cells while others were interacting with the cellular membrane. After 4 h, Synapt-JC-1 were absorbed by the cells. The increase in mitochondrial uptake overtime was confirmed by fluorescence measurements that showed a significant fluorescence intensity inside the cells after 4 h (Fig. 6d). The efficiency of mitochondrial cell delivery, defined as the percentage of live cells receiving mitochondria, was around 40% after 4 h.

As a further check, the presence of specific mitochondrial proteins such as TOM40, Cytochrome c and Hexokinase II was searched in recipient cells after incubation with their specific antibodies. Figure 6e and F show that all three proteins are much more considerable than in control. Finally, to confirm the occurrence of mitochondrial transfer, PCR analysis was performed. As expected, the amount of Rat mtDNA (mitochondrial DNA) significantly increased in LAN5 cells proportionally to the number of synaptosomes administrated, whereas Human mtDNA and nDNA (nuclear DNA) did not change (Fig. 6g). Thus, synaptosomes can be considered a valid delivery system for transplanting healthy mitochondria.

### Restore of mitochondrial homeostasis by mitochondrial transfer

**Fig. 7:**
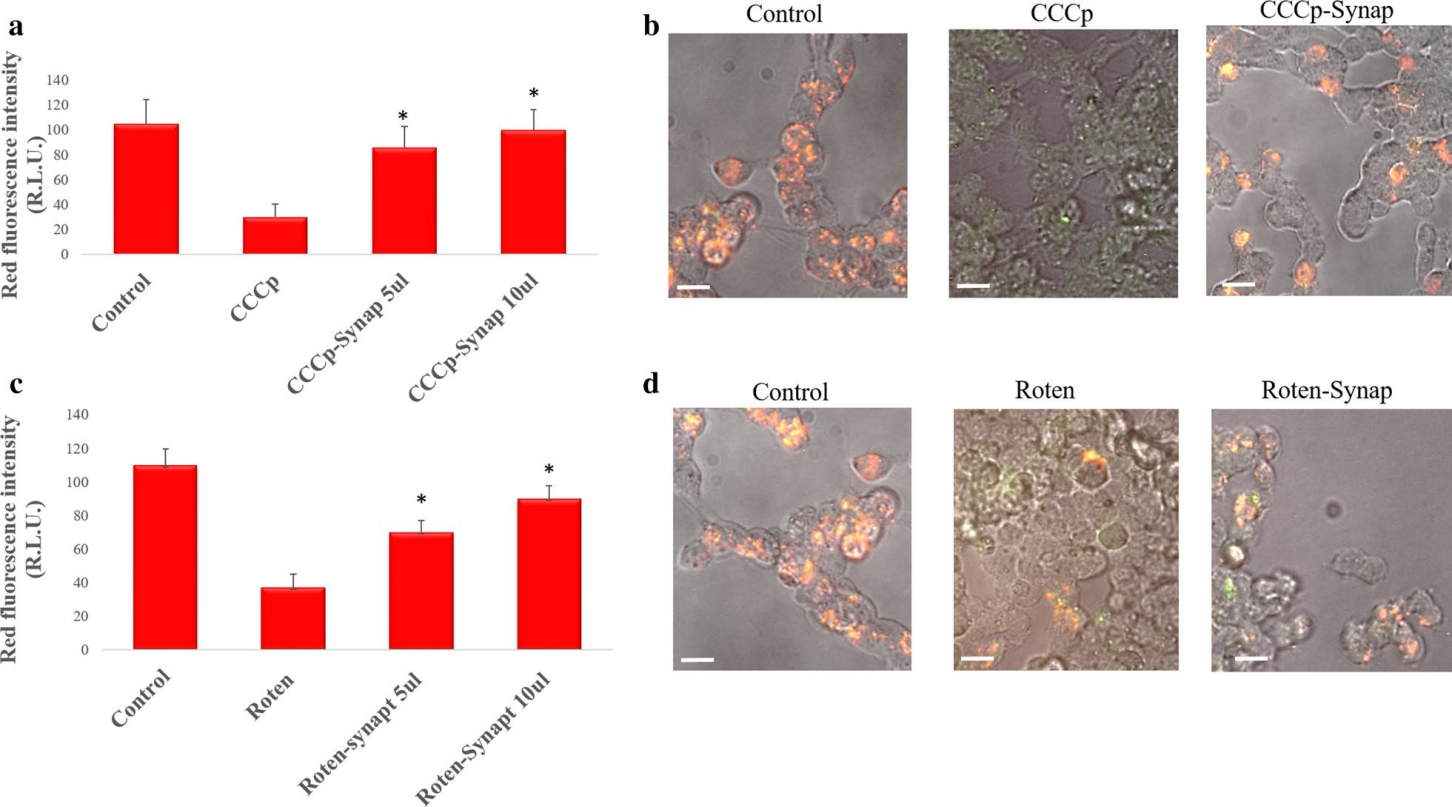
Synaptosomes can replace damaged mitochondria. **a** Histogram of JC1 red fluorescence intensity for LAN5 cell untreated (Control) or treated with CCCp (CCCp) alone or with synaptosomes (CCCp- Synap) at different doses (5 and 10 µl). **b** Representative images of LAN5 untreated (Control) or incubated with CCCp alone or with synaptosomes (CCCp- Synap) after JC-1 assay. **c** Histogram of JC1 red fluorescence intensity for LAN5 cell untreated (Control) or treated with Rotenone (Roten) alone or with synaptosomes (Roten-synap) at different doses (5 and 10 µl). **d** Representative images of LAN5 untreated (Control) or incubated with Rotenone (Roten) alone or with synaptosomes (Roten- Synap) after JC-1 assay. *P < 0.05, *vs* CCCp or Rotenon. Scale bar 20 µm. Synap 5–10 µl, correspond to concentration of 5.1 × 10^7^; 10.2 × 10^7^ particles/100 µl respectively)

We investigated the possibility of replacing damaged with healthy mitochondria within a cell by using synaptosomes as vehicles. To this purpose, we treated LAN5 cells with CCCp or Rotenone, two compounds that induce mitochondrial dysfunction and disruption. After the treatment, red fluorescence emitted by JC1 vital mitochondria was found to decrease with respect to the control, highlighting that mitochondrial function had been inhibited (Fig. 7a, c). Then, synaptosomes were administered in a different dose and re-observed by fluorescence measurements. A dose-dependent increased red fluorescence was observed, indicating that the healthy mitochondria transported by synaptosomes had restored the damaged mitochondrial activity (Fig. 7a, c). The recovery of mitochondrial activity in damaged LAN5 cells after synaptosomes treatment was also confirmed by microscopy fluorescence inspection (Fig. 7b, d).

## Discussion

Mitochondrial dysfunction constitutes the base of pathological events, including neurological diseases [2]. Decreasing of ATP production and endogenous antioxidants, increasing of ROS generation, and alteration of membrane potential are the main characteristics of damaged mitochondria. Although considerable much work has been done to discover therapeutic drugs targeted to mitochondrial dysfunction, so far, no clinical treatment is still available. Mitochondrial transplantation is an innovative technique based on the possibility of replacing damaged mitochondria with healthy exogenous mitochondria [16–18]. The most challenging step in the method is the mitochondria delivery since during the transfer, mitochondria must overcome the transition from an intracellular to an extracellular environment and overcome cell membrane and barriers. Currently applied methodologies for mitochondrial transfer employ naked mitochondria and up to now, no delivery system has been reported. The use of naked mitochondria presents a limit in terms of delivery efficiency due to the possible occurrence of mitochondrial damage and functional alteration during the isolation and transfer processes associated with no specific cellular target [28, 29]. The possibility to encapsulate mitochondria within liposomes has been explored, but some difficulties have been found in organelle packaging [19]. To overcome this problem, it has been recently proposed to use a cell-penetrating peptide (Pep-1) conjugated with mitochondria, and improved uptake by the recipient cells and mitochondrial rescue function were observed [20]. Here, we take evidence that synaptosomes can be a neuronal delivery system of naturally encapsulated mitochondria.

Preparation of Synaptosomes was validated by the presence of Synaptophysin and PSD95 and, consistently with other reports, by intact particles ranging between 0.5–1.5 µm [30]. Further, the high value of the ζ potential and the polydispersity index signified that synaptosomes have good stability, do not tend to flocculate or aggregate and maintain their functional integrity, suggesting that they could be suitable vehicles for the delivery of their cargo, including mitochondria.

An effective delivery system requires an efficient uptake and release of the encapsulated molecules into cells. Nile red or CTX-FITC labelled synaptosomes were internalized into LAN5 recipient cells, as demonstrated by the fluorescence increase and rat synaptophysin presence. Cellular uptake suggests that synaptosomes can be fused with the plasma membrane through a mechanism that appears mediated by specific proteins. After trypsin treatment, indeed, synaptosomes lost their fusion ability. Furthermore, the presence of proteins involved in membrane fusion machineries such as SNARE and SNAP-25 has been demonstrated by the synaptosomal proteomic approach [31]. The specificity of uptake by neuron cells suggests that synaptosomes could contain proteins that act as specific and natural directing agents for the brain. Similar behaviour has been noticed in exosomes released from stimulated cortical neurons that interact selectively with neurons [32]. In addition, the release ability was demonstrated by the presence in LAN5 cellular body of curcumin previously loaded on synaptosomes. Thus, the physical–chemical characteristics, the ability of cell uptake, the cargo release and the specific cell targeting make synaptosomes a natural neuronal delivery system.

The synaptosomes contain mitochondria as demonstrated by the presence of specific mitochondrial proteins such as Opa1, Fis1 and TOM40, and their vitality was confirmed by maintaining of the membrane potential. Furthermore, vital JC1-labeled mitochondria after internalization were released by synaptosomes into the cytosol of neuronal cells, as demonstrated by an increase of fluorescence. Further, an increase of TOM 40, cytochrome C, Hexokinase II proteins and the presence of mitochondrial rat DNA, suggests that synaptosomes could be a vehicle for vital mitochondrial transfer. In addition, the supplement of healthy mitochondria into cells treated with rotenone or CCCp damaged mitochondria was beneficial to restore the mitochondrial function. Hence, synaptosomes can be both the source and the delivery system of mitochondria.

In physiological conditions, mitochondria can be transferred from a donor cell to an injured recipient cell by different contact modes, including tunnelling nanotubes, extracellular vesicles, cell–cell fusion, GAP junctions [33–35]. On the basis of these knowledge, McCully and colleagues employed mitochondrial transplantation as a therapeutic approach to treat ischemia both in animal models and in paediatric patients [18, 36]. Mitochondrial transplantation is reported as a "magical" cure [28], since healthy organelles, harvested by unaffected tissue, after injection in the ischemic hearth move to the injured cells, rescue ATP energy production and improve the contractile function in 10 min [36]. However, this approach, as stated before, present some problems regarding mitochondrial survival during the transfer. Only a small percentage (10%) of the injected mitochondria reaches the target cells and exerts a therapeutic effect [17]. Although mitochondrial supplementation has been mainly utilized for cardiac injuries, this approach has also been applied for the treatment of NDs and other injuries of the nervous system. [19, 29, 37]. In the brain, the use of synaptosomes could improve mitochondrial delivery by protecting the organelle during the transfer, facilitating their specific cellular uptake and reducing stress in the recipient cells. Further, the time of mitochondria transfer could be reduced. The possibility to use synaptosomes as a delivery system for mitochondria agrees with the suggested possibility to encapsulate isolated mitochondria in biomaterials for improving their delivery to the brain and subsequent uptake by cells [37]. In addition, to load antioxidant molecules, such as curcumin, within synaptosomes could be an additional strategy to protect mitochondria by stress occurring during their transfer and/or into the beneficiary cells.

McCully and colleagues indicated that for successful mitochondrial transplantation, vital mitochondria once isolated must be immediately used since, as for their experience, frozen mitochondria do not play their cardioprotection role [36].

Our findings demonstrate that synaptosomes can be cryopreserved for a long time. Indeed, they maintain after thawing their physical–chemical properties and deliver mitochondria vital and physiologically active. Cryopreservation can be a valid strategy in experimental procedures for reducing the number of sacrificed animals and allowing them to do experiments whenever required.

**Fig. 8:**
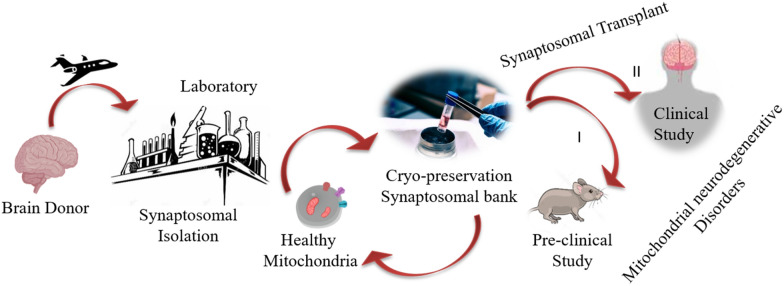
Schematic representation of the step sequence to build a postmortem synaptosomal bank to be used as a reserve of mitochondria for transplantation in pre-clinical and/or clinical studies on neurodegenerative diseases

Nowadays, the brain donation program is one of the most valued contribution to scientific research. Differently from other organ donations, the brain is not used to be transplanted, but half tissue is used for clinic analyses and half is kept, for eventual scientific studies, in a brain bank. Thus, we cannot exclude that in the future, synaptosomes could be extracted from the brain of donors, kept in a synaptosomal-bank and used as a source of mitochondria when necessary for transplantation in mitochondria-damaged neuronal cells (Fig. 8).

This procedure could permit to have an always-available source of functional mitochondria ready to be transplanted, overcoming the necessity to isolate fresh mitochondria and reducing the time before transplantation. In medical practice, indeed, it is necessary to obtain functional mitochondria in a short time because protracting surgeries could be counterproductive. However, for clinical application, the criteria of Good Manufacturing Practice (GMP) have to be established. Quality control of size, number, viability and function of both synaptosomes and organelles must be validated. Furthermore, delivery protocols require to be developed for establishing the number of mitochondria-delivered synaptosomes and the administration route. For cardiac injuries, isolated mitochondria have been transplanted by systemic or in situ injection and both the methods have desirable and undesirable effects [29]. A high number of mitochondria can be transplanted by systemic injection, but they might also be spread in no-injured tissues [31]. By in situ injection, all the isolated mitochondria can be delivered to the target tissue, but side effects due to accumulation of the organelles could occur [38]. Further, the injection could not be repeated if necessary during the surgery. In situ transplantation of mitochondria into CNS has been performed in preclinical studies, but its application in clinic results remains arduous [19]. Recently, the possibility to transplant isolated mitochondria in the damaged brain through the cerebrospinal fluid (CSF) has been explored [39]. Thus, the intraspinal injection could be a new route for transferring mitochondria to the brain and synaptosomes could facilitate the delivery.

## Conclusion

Synaptosomes are not only a source of mitochondria but also a natural delivery system that could improve their neuronal transfer and cellular uptake. Mitochondrial transplantation is a smart strategy to replace or supplement damaged mitochondria. Overall, synaptosome-mediated mitochondrial transplantation could be applicable for the treatment of many brain diseases in which traditional therapies have been unsuccessful.

## Methods

### Synaptosomes isolation from fresh brain tissue

Synaptosomes were isolated by a modification of the method by Franklin et al., (2016) [40]. Briefly, Wistar rats were purchased from Charles River Laboratories (Calco-Lecco, Italy). Explanted rat brains were donated from Palermo University (Italy) in accordance with the authorization number 69636.N.JCO approved by Italian Ministry of Health (Rome, Italy). The cortex (60 mg) was quickly removed and homogenized in 180 µL of 0.32 M sucrose with 10% protease (Amersham Biosciences, Milan, Italy) and phosphatase cocktail inhibitors (cocktail II and III; Sigma-Aldrich, Milan, Italy) with a Dounce on ice. An aliquot (10 μl) was immediately flash-freeze in liquid nitrogen and saved as homogenate.

The homogenate (170 μl) was mixed with 720μL of 2 M sucrose and 300μL of 0.1 mM CaCl2, transferred to 5 ml ultracentrifuge tube and ultracentrifuged at 127,000 RCF for 3 h at 4 °C by using TLA110 rotor (Beckman Coulter, Brea, CA). Successively, the myelin layer floating on the top was discarded, and the synaptosomal band at the sucrose layers interface was collected (Fig. 1b). Then, the synaptosomal band was transferred into an ultracentrifuge tube and centrifuged at 18,200 RCF for 30 min at 4 °C in TLA110 rotor (Beckman Coulter, Brea, CA). The supernatant was discarded and the pellet, containing the synaptosomes, was resuspended in 5 ml of HBK buffer (HEPES-buffered Krebs-like) (143 mM NaCl, 4.7 mM KCl, 1.3 mM MgSO_4_, 1.2 mM CaCl2, 20 mM HEPES, 0.1 mM NaH_2_PO_4_ e 10 mM D-glucose a pH 7.4) to be analyzed.

For cryopreservation, the synaptosomal pellet was suspended in 5 ml HBK buffer containing 10% Fetal Bovine Serum (FBS) (Gibco-Invitrogen, Milan, Italy), 10% DMSO, split into 5 aliquots (1 ml) and cryopreserved by performing a sequence of controlled freezing steps consisting in10 min at 4 °C, overnight at − 20 °C, 24–48 h at − 80 °C. Finally, the aliquots were stored in liquid nitrogen.

### Zeta potential, concentration and polydispersity index

Fresh or thawed Synaptosomal aliquots were resuspended in 2.5 ml HBK and diluted (1:100) in PBS 1X. The zeta potential, concentration (particles/ml) and polydispersity index were measured using the instrument ZetaView® BASIC NTA—Nanoparticle Tracking Video Microscope PMX-120.

### Quasi-elastic laser light scattering (QELS)

Fresh or thawed Synaptosomal aliquots were resuspended in 2.5 ml of HBK (5.1 × 10^9^ ± 0.7 × 10^9^ particles/ml) and QELS experiments were carried out at T 25 °C after synaptosomes dilution (1:10) in HBK at pH 7.4. The cuvette was placed in the thermostatically controlled cell compartment of a Brookhaven Instrument BI200-SM goniometer equipped with a 15 mW He-Neon Spectra Physics laser tuned at λ 632.8 nm. The temperature was controlled within 0.05 °C by a circulating bath. Scattered light intensity at 90°, I90°(t), and its time autocorrelation function, g2(t), were measured by using a Brookhaven BI-9000 correlator. Autocorrelation functions g2(t) were analyzed using a smoothing constrained regularization method CONTIN [41] to obtain the intensity weighted diameter distribution of synaptosomes.

### Atomic Force Microscopy (AFM)

Fresh or thawed synaptosomal aliquots were resuspended in 2.5 ml HBK (5.1 × 10^9^ particles/ml) and diluted (1:1000). 50 μl of the synaptosome suspension were deposited on freshly cleaved mica, washed after few minutes and dried under a mild vacuum. Tapping mode AFM images were acquired in the air using a multimode scanning probe microscope driven by a Nanoscope V controller (Digital Instruments, Bruker). Single beam uncoated silicon cantilevers (type SPM Probe Mikromasch) were used. The drive frequency was between 260 and 325 kHz; the scan rate was 0.25–0.7 Hz.

### Cell cultures and treatments

LAN5 neuroblastoma, HepG2 human liver cancer and A549 adenocarcinomic human alveolar basal epithelial cell lines, were cultured with RPMI 1640 medium (Celbio srl, Milan, Italy) supplemented with 10% FBS (Gibco-Invitrogen, Milan, Italy), 2 mM glutamine and 1% penicillin, 1% streptomycin (50 mg/ml) (Sigma). The cells were treated with synaptosomes (5.1 × 10^9^ particles/ml), at different dilutions 5–10–20 µl in 100 µl of cell medium for 2, 4 or 24 h (corresponds to concentration of 2.5 × 10^7^; 5.1 × 10^7^; 10.2 × 10^7^ particles/100 µl respectively).

In transplantation experiments, cells were pre-treated with 40 µM rotenone for 1 h and 50 µM of Carbonyl cyanide 3- chlorophenylhydrazone (CCCp) for 5 min at 37 °C. After washing, cells were incubated without or with synaptosomes at different dilutions (5-10 µl in 100ul of the medium) for 24 h at 37 °C. When indicated, synaptosomes were treated with 1X trypsin (Sigma) or with 50 mM CCCp for 5 min.

### Synaptosomes labeling with membrane fluorescence dyes

Fresh or thawed synaptosomal aliquots were resuspended in 2.5 ml HBK. 500 µl of the synaptosome solution (5.1 × 10^9^ particles/ml) were stained by the membrane dye Nile Red or fluorescein-tagged cholera toxin (CTX-FITC). Nile Red is used to stain lipids since it is highly fluorescent in a non-polar environment (excitation/emission ~ 490/590). CTx-FITC stains lipid rafts (excitation/emission maxima 490/535). For staining, synaptosomes were incubated in HBK buffer with Nile Red (5 µg/ml) for 15 min and with CTX-FITC (10 µg/ml) for 30 min at 37 °C in the dark. After incubation, synaptosomes were centrifuged at 10,000 RCF for 10 min and then washed two times with HBK to remove the dye excess. Fluorescence emission of synaptosomes marked with Nile Red or CTx-FITC was measured by fluorimeter (Glomax) and fluorescence microscopy (Zeiss).

### Cellular uptake of Synaptosomes

For uptake studies, LAN5, HepG2, and A549 cells were plated at the concentration of 3 × 10^5^ cell/ml in 96-well plate. The cells were incubated with 20 µl of synaptosomes (10.2 × 10^7^ particles/100 µl), labeled with Nile Red for 4 h. After washing with PBS, the incorporated fluorescence was measured by a fluorescence microscope (Axio Scope 2 microscope; Zeiss) or fluorimeter (Glomax). The levels of fluorescence for the cell was analyzed by Image J software to compare the fluorescence intensity between the different cell lines.

### Synaptosomes release assay

To test the ability of synaptosomes to release molecules into cells, we used curcumin, a natural fluorescent compound. Fresh or thawed synaptosomal aliquots were resuspended in 2.5 ml of HBK and curcumin loading was performed by incubating 500 µl of synaptosomes (5.1 × 10^9^ particles/ml) with curcumin (5 µM) overnight at room temperature. Then, the excess of curcumin in the sample was removed by centrifugation at 10,000 RCF for 10 min and two washes with HBK. The amount of curcumin incorporated in synaptosomes was measured by using a fluorimeter (Glomax) and fluorescence microscopy (Zeiss). LAN5 cells were plated at the concentration of 3 × 10^5^ cell/ml in a 96-well plate and incubated with 10 or 20 µl curcumin-loaded synaptosomes (5.1 × 10^7^; 10.2 × 10^7^ particles/100 µl) for 2 and 4 h. After treatment, cells were washed with PBS, and the emitted fluorescence was evaluated by fluorescence microscope (Axio Scope 2 microscope; Zeiss) or fluorimeter (Glomax) Excitation/emission maxima ~ 490/535.

### Analysis of mitochondrial activity in synaptosomes

Membrane potential of synaptosomal mitochondria was measured using MitoProbe JC-1 assay kit (Thermo Fisher Scientific–US). JC-1 (5,50,6,60-tetrachloro- 1,10,3,30-tetraethylbenzimidazolyl-carbocyanine iodide) is a dye that enters into mitochondria and changes colour from green to red as the membrane potential increases. Fresh or thawed synaptosomal aliquots were resuspended in 2.5 ml of HBK and 100 µl were plated in a 96-well plate. After the addition of JC-1 (2 µM), the sample was incubated for 30 min at 37 ºC. CCCp (50 µM), a mitochondrial membrane potential disrupter, was used as control. The excitation length for JC-1 is 490 nm, and red and green emission lengths are 590 and 529 nm, respectively.

### Synaptosomal mitochondria delivery

Fresh or thawed synaptosomal aliquots were resuspended in 2.5 ml of HBK and 500 µl (5.1 × 10^9^ particles/ml) were incubated with 2 µM JC-1 (Thermo Fisher Scientific–US) for 30 min at 37 °C in the dark, and then washed two times in PBS to remove the dye excess. LAN5 cells were plated at the concentrations of 3 × 10^5^ cell/ml in a 96-well plate and incubated with synaptosomes containing JC-1 marked mitochondria for 2 and 4 h. After the treatment, cells were washed with PBS, and the fluorescence was evaluated by fluorescence microscope (Axio Scope 2 microscope; Zeiss). The excitation and emission lengths were 490 and 590 nm, respectively. The levels of fluorescence for the cell was analyzed by Image J software.

### Total protein extraction and Western blotting

Total proteins were extracted by dissolving cells and synaptosomes in the solubilizing buffer (50 mM Tris–HCl pH 7.4, 150 mM NaCl, 0.5% Triton X-100, 2 mM phenylmethylsulphonyl fluoride PMSF,1mMDTT, 0.1% SDS) with protease inhibitor (Amersham Biosciences, Milan, Italy) and phosphatase inhibitor (cocktail II and III; Sigma Aldrich, Milan, Italy). Protein samples (30 µg) were submitted to 10% SDS PAGE and transferred onto nitrocellulose filters. The Western blot was incubated with anti-TOM40 (1:1000; Cell Signaling, Boston, USA), anti-Cytochrome C (1:1000; Cell Signaling, Boston, USA), anti-OPA1 (1:1000; Cell Signaling, Boston, USA), anti-FIS1(1:1000; Cell Signaling, Boston, USA), anti-synaptophysin (1:2000; Cell Signaling, Boston, USA), PSD95(1:1000; Cell Signaling, Boston, USA), anti-βActin (1:1000; SIGMA) antibodies. Primary antibody was detected by the Odyssey scanner (L-Licor) and using secondary antibody labeled with IR 790, (1:10,000; Life Technology) according to the manufacturer's instructions. Band intensities were analyzed with ImageJ and expression was adjusted to βActin expression. The protein levels were expressed as intensity relative to control.

### Statistical analysis

All experiments were repeated three times, and in each experiment, the samples were taken in triplicate. The results are presented as mean ± SD. Statistical evaluation was conducted by ANOVA, for analysis of significance. Results with a P value p ≤ 0.05 were considered statistically significant, *P ≤ 0.05, **P ≤ 0.01.

## Supplementary Information

**Additional file 1: Figure S1.** A) Morphological analysis of LAN5 cells incubated with different doses (5-10 and 20 µl, which correspond to concentration of 2.5 × 107; 5.1 × 107; 10.2 × 107 particles/100µl respectively) of synaptosomes (Synap). B) Nuclear staining by fluorescence probe Hoechst 3341 of LAN5 cells incubated with different doses (5-10 and 20 µl) of synaptosomes (Synap).

## Data Availability

The datasets used and/or analyzed during the current study are available from the corresponding author on reasonable request.
