# Supplementary figures and images for: Synaptosomes: new vesicles for neuronal mitochondrial transplantation
Source: J Nanobiotechnology. 2021 Jan 6;19:6. doi: 10.1186/s12951-020-00748-6 (PMC7789323; doi:10.1186/s12951-020-00748-6)

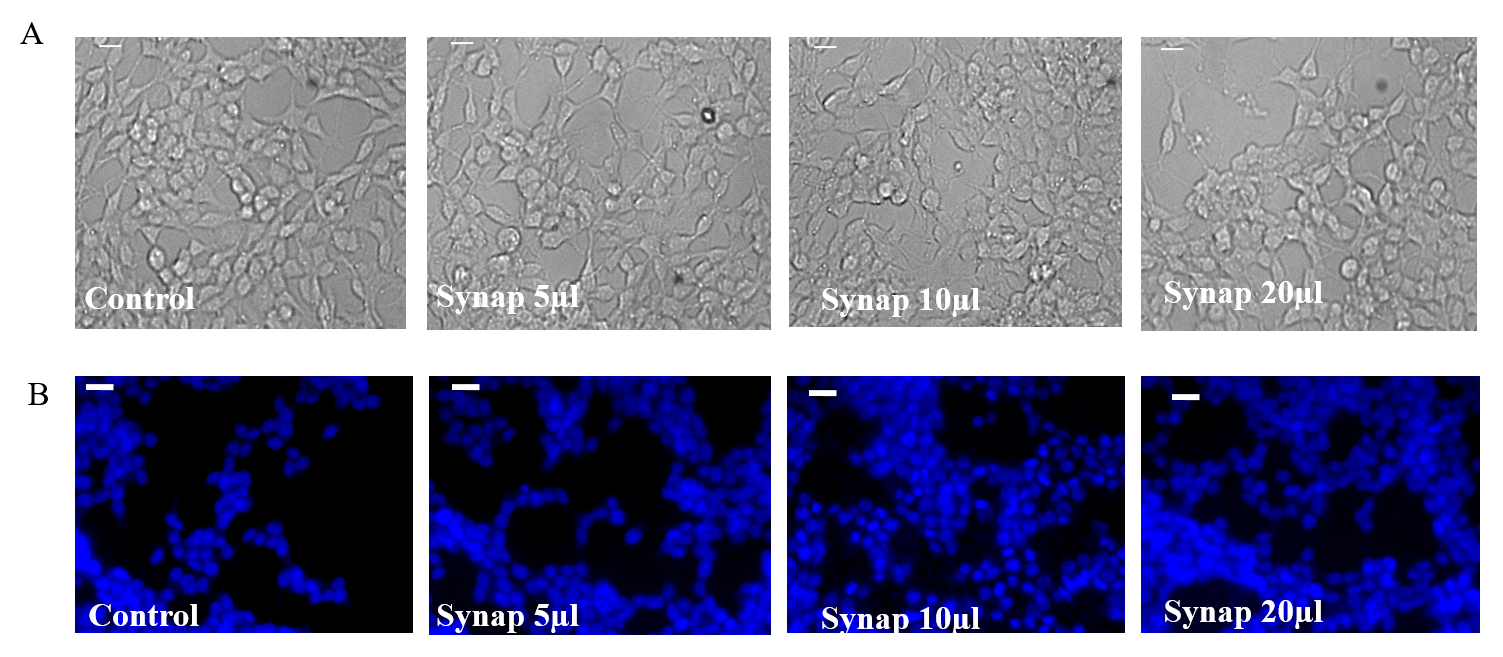

Supplement: Supplementary file 1 — Additional file 1: Figure S1. A) Morphological analysis of LAN5 cells incubated with different doses (5-10 and 20 µl, which correspond to concentration of 2.5 × 10^7^; 5.1 × 10^7^; 10.2 × 107 particles/100µl respectively) of synaptosomes (Synap). B) Nuclear staining by fluorescence probe Hoechst 3341 of LAN5 cells incubated with different doses (5-10 and 20 µl) of synaptosomes (Synap). [file 12951_2020_748_MOESM1_ESM.tif]
